# Lysine Demethylases: Promising Drug Targets in Melanoma and Other Cancers

**DOI:** 10.3389/fgene.2021.680633

**Published:** 2021-06-16

**Authors:** Gaya Punnia-Moorthy, Peter Hersey, Abdullah Al Emran, Jessamy Tiffen

**Affiliations:** ^1^Melanoma Oncology and Immunology Group, Centenary Institute, University of Sydney, Sydney, NSW, Australia; ^2^Melanoma Epigenetics Laboratory, Centenary Institute, University of Sydney, Sydney, NSW, Australia; ^3^Melanoma Institute Australia, University of Sydney, Sydney, NSW, Australia

**Keywords:** epigenetics, histones, lysine demethylases, small molecule inhibitors, cancer, melanoma

## Abstract

Epigenetic dysregulation has been implicated in a variety of pathological processes including carcinogenesis. A major group of enzymes that influence epigenetic modifications are lysine demethylases (KDMs) also known as “erasers” which remove methyl groups on lysine (K) amino acids of histones. Numerous studies have implicated aberrant lysine demethylase activity in a variety of cancers, including melanoma. This review will focus on the structure, classification and functions of KDMs in normal biology and the current knowledge of how KDMs are deregulated in cancer pathogenesis, emphasizing our interest in melanoma. We highlight the current knowledge gaps of KDMs in melanoma pathobiology and describe opportunities to increases our understanding of their importance in this disease. We summarize the progress of several pre-clinical compounds that inhibit KDMs and represent promising candidates for further investigation in oncology.

## Introduction

There are numerous genetic alterations which promote carcinogenesis which include mutations of certain genes and chromosomes. Epigenetics is defined as heritable functional changes in the genome which do not involve a change in the DNA sequence (Dupont et al., 2009). The Greek prefix “epi” denotes “over, outside of, or around” implying additional factors that may influence traditional genetic inheritance patterns. Epigenetics is essential in normal development and biology but dysregulation has been implicated as a key impetus of carcinogenesis and resistance. All cells contain genetic information in the form of DNA which is wound around proteins called histones (Kornberg, 1974). The DNA is assembled into units called nucleosomes which form a complex consisting of histones and DNA known as chromatin (Kornberg, 1974). Chromatin is organized into compact, transcriptionally inactive regions called heterochromatin, usually around the periphery of the nucleus and loosely arranged, transcriptionally active chromatin called euchromatin (Elgin, 1996). This formation has an important role in the regulation of gene expression as well as controlling the transcription, replication, recombination and repairing of DNA (Elgin, 1996). Various epigenetic changes can affect chromatin structure and hence gene expression. One of the most well studied epigenetic changes involves methylation which may occur at the DNA or histone level (Tost, 2010). DNA methylation involves the transfer of methyl groups by DNA methyltransferases (DNMTs) to individual nucleotide bases, altering gene expression (Bilian Jin and Robertson, 2011). The family of DNMT enzymes adds methyl groups while removal is mediated by the ten eleven translocation (TET) family (Zhang et al., 2010).

**FIGURE 1:**
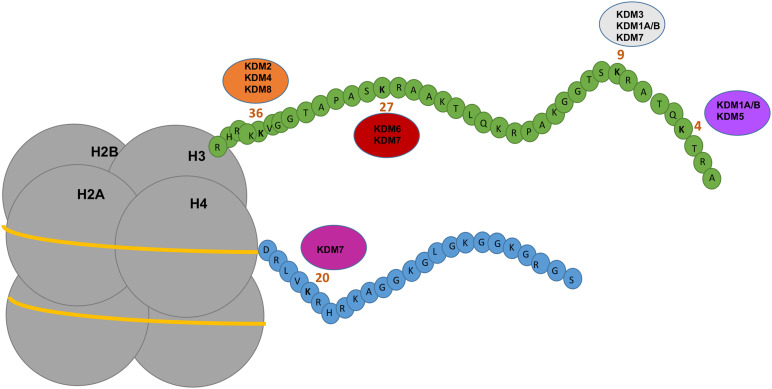
Depicting the mechanism of action of KDMs on histones. Nucleosomes consist of four subunits of histones which contain amino acid tails that are modified by epigenetic regulators including KDMs. KDM1A/B demethylates H3K9me1/H3K9me2 and H3K4me1/me2 inducing gene repression or activation. KDM2 and KDM4 demethylases target H3K36me1/me2/me3 inducing gene activation. KDM3 demethylases target H3K9me1/me2 inducing gene activation. KDM5 demethylases targets H3K4me2/me3 inducing gene repression. KDM6 and KDM7 demethylases target H3K27me2/me3 inducing gene activation. KDM7 demethylases target H4K20me1 and H3K9me2/me3. KDM8 demethylase target H3K36me3 inducing gene activation. Figure is adapted from Verde et al. (2017).

The other major class of methylation is histone methylation which is a post-translational modification (PTM) of histone tails. Histone methylation involves the addition of (*via* writer enzymes) or removal of (*via* eraser enzymes) methyl groups, typically on lysine (K) or arginine (R) amino acids of histone type 1–4 tails (Figure 1). Lysine methyltransferases (KMTs) drive the addition of methyl groups, whereas lysine demethylases (KDMs) are responsible for the removal of methyl groups. Depending on the type of histone modifications, the consequence might induce either an open or closed chromatin state which regulates gene expression. KDMs rarely act in isolation as enzymatic erasers but are typically members of large epigenetic complexes, consisting of other enzymes and transcription factors. This is suggestive of a scaffolding protein role for KDMs in addition to a catalytic one. The most well investigated histone lysine methylation sites include H3K4, H3K9, H3K27, H3K36, H3K79, and H4K20 (Figure 1) that will either compact or open chromatin depending on lysine position and the number of methyl groups. Hence this review will focus on what is currently known about KDMs in normal biology and cancers including melanoma. A summary of the progress of current KDM inhibitors is also discussed.

## The Function of KDMs in Normal Development and Deregulation in Cancer

Lysine demethylases are critical for normal development but numerous studies have implicated the dysregulation of several KDMs in cancers. This is likely due to the ability of KDMs to govern major changes in the transcriptional networks of hundreds of genes with potential roles in all major hallmarks of cancer. The roles of each KDM in normal biology and cancer is described below and Table 1 summarizes studies in cancer.

**TABLE 1:** Summary of KDMs reported to be significant in cancer.

KDM	Alias	Target Histone demethylation site	Gene activation or repression	Cancer study implicated in
KDM1A	LSD1, AOF2, BHC110, KDM1	H3K4me1 H3K4me2	Repression Repression	Leukemia (Harris et al., 2012), prostate cancer (Willmann et al., 2012), breast cancer (Perillo et al., 2008), neuroblastoma (Schulte et al., 2009; Amente et al., 2015)
KDM1B	LSD2, AOF1	H3K4me1 H3K4me2	Repression Repression	Breast cancer (Katz et al., 2014; Chen et al., 2017)
KDM2A	JHDM1A, FBXL11	H3K36me1 H3K36me2	Activation Activation	Leukemia (Dong et al., 2013), non-small cell lung cancer (NSCLC) (Wagner et al., 2013), gastric cancer (Huang et al., 2015; Kong et al., 2016), breast cancer (Rizwani et al., 2014)
KDM2B	JHDM1B, FBXL10	H3K36me1 H3K36me2 H3K4me3	Activation Activation Repression	Leukemia (He et al., 2011), pancreatic cancer (Tzatsos et al., 2013), ovarian cancer (Kuang et al., 2017), gastric cancer (Zhao et al., 2017), glioma (Wang Y. et al., 2018)
KDM3A	JHDM2A, JMJD1A, JMJD1	H3K9me1 H3K9me2	Activation Activation	Breast cancer (Ramadoss et al., 2017a), ovarian cancer (Ramadoss et al., 2017b), Ewing sarcoma (Sechler et al., 2017), prostate cancer (Wilson et al., 2017)
KDM3B	JHDM2B, JMJD1B	H3K9me1 H3K9me2	Activation Activation	Leukemia (Kim et al., 2012)
KDM3C	JHDM2C	H3K9me1 H3K9me2	Activation Activation	unknown
KDM4A	JMDM3A, JMJD2A	H3K9me2 H3K9me3	Activation Activation	Endometrial cancer (Qiu et al., 2015), breast cancer (Berry et al., 2012)
		H3K36me2 H3K36me3	Activation Activation	
KDM4B	JMDM3B, JMJD2B	H3K9me2 H3K9me3	Activation Activation Activation Activation	Breast cancer (Kawazu et al., 2011) Colorectal cancer (Li et al., 2020)
		H3K36me2 H3K36me3		
KDM4C	JMDM3C, JMJD2C	H3K9me2 H3K9me3	Activation Activation	Prostate cancer (Wissmann et al., 2007)
		H3K36me2 H3K36me3	Activation Activation	
KDM4D	JMDM3D, JMJD2D	H3K9me3	Activation	Prostate cancer (Shin and Janknecht, 2007)
KDM4E	KDM4DL, JMJD2E	H3K9me3	Activation	Unknown
KDM5A	JARID1A RBBP2	H3K4me2 H3K4me3	Repression Repression	Leukemia (van Zutven et al., 2006),breast cancer (Hou et al., 2012), ovarian cancer (Feng et al., 2017), melanoma (Roesch et al., 2005)
KDM5B	JARID1B PLU1	H3K4me2 H3K4me3	Repression Repression	Breast cancer (Catchpole et al., 2011), prostate cancer (Xiang et al., 2007), melanoma (Roesch et al., 2010)
KDM5C	JARID1C SMCX	H3K4me2 H3K4me3	Repression Repression	Cervical cancer (Smith et al., 2010), renal cell carcinoma (Yan et al., 2007; Niu et al., 2012)
KDM5D	JARID1D	H3K4me2 H3K4me3	Repression Repression	Prostate cancer (Komura et al., 2016, 2018; Li et al., 2016)
KDM6A	UTX	H3K27me2 H3K27me3	Activation Activation	Bladder cancer (Ler et al., 2017), cervical cancer (Soto et al., 2017), breast cancer (Taube et al., 2017), multiple myeloma (Ezponda et al., 2017), lung cancer (Terashima et al., 2017), pancreatic cancer (Andricovich et al., 2018)
KDM6B	JMJD3	H3K27me2 H3K27me3	Activation Activation	Colon cancer (Pereira et al., 2011; Tokunaga et al., 2016), pancreatic cancer (Yamamoto et al., 2014), Prostate cancer (Daures et al., 2016), diffuse large B-cell lymphoma (Mathur et al., 2017), non-small cell lung cancer (Ma et al., 2015), clear cell renal carcinoma (Li et al., 2015), multiple myeloma (Ohguchi et al., 2017), acute myeloid leukemia (Li et al., 2018), melanoma (Park et al., 2016), ovarian cancer (Pinton et al., 2018)
KDM7A	JHDM1D	H3K27me1 H3K27me2	Activation Activation Activation Activation Activation	Melanoma and cervical cancer (Osawa et al., 2011), Prostate cancer (Lee et al., 2018)
		H3K9me1 H3K9me2 H4K20me2		
KDM8	JMJD5	H3K36me3	Activation	Breast cancer (Hsia et al., 2010) Prostate cancer (Wang et al., 2019)

### Amine Oxidase KDMs

Lysine demethylases are classified into two groups according to the catalytic mechanisms of demethylation- amine oxidase or jumonji C domain containing KDMs. Class I is the amine-oxidase lysine specific demethylases 1 and 2 (LSD1 and 2), also known as KDM1A and KDM1B. KDM1A and KDM1B use Flavin adenine dinucleotide (FAD) as a substrate to generate an imine intermediate which is hydrolyzed to produce the demethylated lysine residue (Walport et al., 2012). The amines-oxidase like (AOL) catalytic domain at the C-terminal in KDM1A and KDM1B consists of two folded subdomains. The FAD and substrate binding regions are structurally related to the superfamily of monoamine oxidases (MAO). In particular MAO-A and MAO-B- enzymes which catalyze the oxidation of monoamine (contain one amino group) neurotransmitters which include serotonin and dopamine (Shi et al., 2004; Willmann et al., 2012). The N-terminal of KDM1A contains the SWIRM domain which is named after the SWI3, RSC8 and MOIRA proteins and is essential for protein stability and interactions with histone tails.

KDM1A was the first histone demethylase identified (Shi et al., 2004). Functionally, KDM1A predominantly catalyzes the removal of methyl groups from mono and di-methylated lysine residues at H3K4 inducing gene repression (Walport et al., 2012). An early *in vivo* study showed that KDM1A demethylation of K1096 in the DNA methyl transferase enzyme DNMT1 was essential for the gastrulation stage of murine embryogenesis (Wang et al., 2009). Genetic ablation of KDM1A in a knockout mouse model was embryonic lethal (Wang et al., 2007, 2009).

Interestingly, KDM1A can demethylate non-histone proteins in osteosarcoma cells such as the tumor suppressor p53 which has an important role in regulation of pro-apoptotic genes. Another example showed KDM1A can demethylate K185 of transcription factor E2F1 which has an important role in regulating the cell cycle and tumor suppressor genes in lung cancer cells (Huang et al., 2007; Wang et al., 2009; Kontaki and Talianidis, 2010; Xie et al., 2011). A study found that overexpression of KDM1A induces E2F1 signaling *via* histone demethylation and promotes cell proliferation in oral cancer and that inhibition of KDM1A reduces E2F1 signaling, implying an oncogenic role of KDM1A in oral cancer (Narayanan et al., 2015).

The other KDM1 family member, KDM1B has been reported to catalyze the removal of methyl groups at H3K4 and this enzyme is essential for oocyte development (Ciccone et al., 2009). KDM1B knockout mice exhibit embryonic lethality (Ciccone et al., 2009), highlighting its importance in normal biology.

### Jumonji C Demethylases

Class II KDMs are the demethylases that contain a Jumonji C domain (JMjC KDMs “Jumonji” meaning cruciform in Japanese). This includes the KDM2-6 subfamilies which consists of 20 enzymes that are grouped into five subfamilies: KDM2, KDM3, KDM4, KDM5, and KDM6 (Klose et al., 2006). The JMjC KDMs catalyze the removal of methyl groups from mono, di and trimethylated lysines at various sites and use dioxygen and 2-oxoglutarate (2OG) as substrates with Fe (II) as an essential cofactor (Walport et al., 2012). Unlike KDM1A/1B the JMjC KDMs are able to remove methyl groups from trimethylated lysine sites since the mechanism doesn’t require a formation of an imine. The catalytic domain of the JMjC KDMs is structurally related to the superfamily of 2OG- dependent oxygenases which play an important role in fatty acid metabolism, protein biosynthesis and nucleic acid repair/modification (Loenarz and Schofield, 2008).

### KDM2/3 Demethylases

The KDM2 subfamily consists of two demethylases KDM2A and KDM2B. Both KDM2A and KDM2B have been reported to be oncogenic (Pedersen and Helin, 2010). KDM2A has been reported to be upregulated and induces proliferation in lung, gastric and breast cancer. KDM2A overexpression was found to increase cell proliferation and invasion through activation of ERK1/2 signaling in lung cancer as well as being associated with poor prognosis in lung cancer patients (Wagner et al., 2013). KDM2A overexpression also promoted cell growth and migration in gastric cancer by downregulation of tumor suppressor gene programmed cell death 4 (PDCD4) (Huang et al., 2015). In breast cancer, KDM2A is highly expressed in myoepithelial cells which have been reported to have anti-tumor properties. KDM2A ablation in these cells induced increased invasion and migration *via* downregulation of MMP proteins and repression of EF21 signaling (Rizwani et al., 2014).

KDM2B is overexpressed in numerous cancers. Knockdown of KDM2B reduced cell growth in gastric cancer *in vitro* and *in vivo* and induced autophagy- a process in which cells remove damaged cell components (Zhao et al., 2017). KDM2B overexpression induces transformation of hematopoietic progenitor cells in acute myeloid leukemia whereas reduction of KDM2B inhibited Hox9/Meis1 induced leukemic transformation (He et al., 2011). Overexpression of KDM2B is observed in ovarian cancer and when knocked down *in vitro* and *in vivo* reduced cell proliferation, migration and tumor growth (Kuang et al., 2017). Pancreatic cancers with increased KDM2B promoted tumor formation in cooperation with the oncogene Kras in an *in vivo* model (Tzatsos et al., 2013). Another study showed that KDM2B overexpression was associated with poor prognosis in glioma and KDM2B knockdown inhibited cell proliferation and induced cell cycle arrest (Wang Y. et al., 2018).

The KDM3 subfamily consists of three demethylases; KDM3A, KDM3B, and JMJD1C. It has been reported that KDM3A is important in spermatogenesis and male KDM3A knockout mice are infertile (Pedersen and Helin, 2010). The function of KDM3B and JMJD1C is largely unknown.

### KDM4/5 Demethylases

The KDM4 subfamily consists of five demethylases- KDM4A, KDM4B, KDM4C, KDM4D, and KDM4E. KDM4A, KDM4B, KDM4C, and KDM4D have been reported to be important in oncogenesis, but the function of KDM4E is unknown (Pedersen and Helin, 2010). KDM4A overexpression was found to stimulate the AR, inducing the expression of prostate specific antigen, implicated in the progression of prostate cancer (Kim et al., 2016). KDM4A is overexpressed in breast cancer and KDM4A knockdown inhibited cell proliferation, migration and invasion (Li et al., 2012). Lung cancers with increased KDM4A are associated with poor prognosis (Xu et al., 2016).

KDM4B overexpression promoted DNA damage in breast cancer cells which was significantly reduced upon pharmacological inhibition of KDM4B, by induction of apoptosis in triple negative breast cancers deficient in the tumor suppressor gene, PTEN (Wang W. et al., 2018; Xiang et al., 2019). KDM4B overexpression also promoted proliferation, growth and glucose uptake in colorectal cancer cells whereas KDM4B knockdown inhibited tumor growth significantly in an *in vivo* model (Li et al., 2020).

KDM4C overexpression is associated with poor prognosis in prostate cancer and can co-regulate transcriptional activation of the AR. KDM4C knockdown significantly reduced proliferation, colony formation, AR transcriptional activity in prostate cancer cells and inhibited tumor growth of a prostate cancer model in zebrafish (Lin et al., 2019).

The KDM5 subfamily consists of four demethylases- KDM5A, KDM5B, KDM5C, and KDM5D. This group of demethylases can remove methyl groups from di and trimethylated lysines on H3K4. The KDM5 subfamily has been reported to play an important role in development, identified in *drosophila melanogaster* as Lid (Little Imaginal Disks) protein due to the phenotype visible in mutant larvae (Gildea et al., 2000). This protein was classified as a H3K4 histone demethylase which had all the domains of the human JARID1 family. The Lid protein binds to *drosophila* Myc (dMyC)—a transcription factor that is important in cell cycle progression, cell proliferation and apoptosis, and is frequently dysregulated in cancer (Secombe and Eisenman, 2007; Li et al., 2010).

KDM5A was initially identified as a binding partner of retinoblastoma protein (pRB) (Defeo-Jones et al., 1991). pRB is a tumor suppressor that inhibits cell cycle progression, preventing cell growth and promoting senescence. Embryonic fibroblasts isolated from KDM5A knockout mouse revealed an important role for KDM5A in mitochondrial function (Varaljai et al., 2015) and cell differentiation (Benevolenskaya et al., 2005; Lopez-Bigas et al., 2008).

KDM5B/JARID1B/PLU1 was initially identified in a study targeting genes regulated by the tyrosine kinase HER2 (Lu et al., 1999). A later study found that KDM5B had H3K4 histone demethylase activity and inhibited the expression of tumor suppressor genes BRAC1 and CAV1 (Yamane et al., 2007). In addition, KDM5B has been reported to be overexpressed in numerous cancers and has been identified as a potential oncogene.

KDM5D/JARID1D is the least well investigated demethylases from the KDM5 subfamily, but has been implicated in prostate cancer progression (Perinchery et al., 2000).

### KDM6/7 Demethylases

**FIGURE 2:**
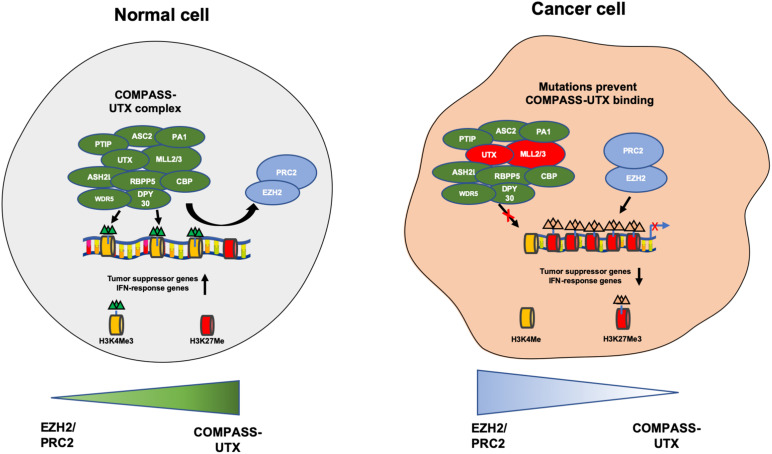
Depicting the COMPASS complex and PCR2 complex that contribute to the open and closed chromatin states. In a normal healthy cell the COMPASS complex removes methyl groups from H3K27me3 and adds methyl groups to H3K4me and prevents PRC2 complex from adding methyl groups to H3K27me. This induces transcriptional activation of tumor suppressive and IFN-response genes. In the context of cancer UTX (KDM6A) and MLL2/3 are frequently mutated in the COMPASS complex, causing a loss of expression. This prevents UTX from binding to the compass complex and no H3K27me3 demethylation occurs, enabling the PRC2 complex to add methyl groups on H3K27me and remove methyl groups from H3K4me, inducing transcriptional repression of tumor suppressor and IFN response genes, promoting cancer cell growth.

The KDM6 subfamily consists of three demethylases- KDM6A, KDM6B, and UTY. KDM6A also known as UTX can remove methyl groups from di and trimethylated lysines on H3K27- a mark associated with suppression of gene transcription (Pedersen and Helin, 2010). KDM6A was initially identified as playing an important role in embryonic development and cell differentiation (Hong et al., 2007). KDM6A together with methyltransferases MLL2 (KMT2D) and MLL3 (KMT2C) is an important component of the COMPASS complex also known as the ASCOM complex which mediates the transcriptional activation of genes *via* H3K4 trimethylation and H3K27me2/3 demethylation (Shilatifard, 2008; Van der Meulen et al., 2014; Ford and Dingwall, 2015). The COMPASS complex can also promote histone *acetylation* a demethylation independent activity, by interaction with histone acetyltransferase p300 (CBP) as well as chromatin remodeling *via* the SWI/SNF complex and transcriptional elongation by interacting with transcription elongation factors (Figure 2; Wang et al., 2017; Schulz et al., 2019). KDM6A therefore has dual roles in activation of gene expression. Not only does it remove suppressive marks on H3K27 but also activates genes by H3K4 trimethylation and H3K27 acetylation, highlighting the complex interplay between histone erasers and writers to remodel chromatin in a highly orchestrated fashion.

KDM6A exhibits another methylation independent role by directly interacting with DNA binding transcription factors, including nuclear receptors such as estrogen and retinoic acid receptors (Cho et al., 2007; Rocha-Viegas et al., 2014; Xie et al., 2017). KDM6A has been found to interact with retinoblastoma binding proteins including RBBP5 *in vivo*, potentially having an influence on the regulation of cell cycle and cell differentiation by RB family proteins (Shpargel et al., 2012; Van der Meulen et al., 2014).

KDM6A has been reported to be inactivated by mutations in 70% of non-invasive bladder cancer causing a loss of KDM6A expression (Ler et al., 2017). This is suggestive of a tumor suppressor role in this cancer. Studies have suggested loss of KDM6A may amplify PRC2 complex mediated gene repression and dependency in bladder cancer cells that can be sensitized to EZH2 inhibitors (Atala, 2017). Loss of KDM6A expression has also been associated with other cancers such as multiple myeloma (MM) in which KDM6A mutations lead to low KDM6A expression, resulting in increased proliferation, adhesion and tumorigenicity. Loss of KDM6A also sensitized MM cells to EZH2 inhibitors GSK343 and GSK126, inducing cell death and decreased proliferation (Ezponda et al., 2017). Squamous cell, metastatic pancreatic cancer in females was also associated with loss of KDM6A expression in a knockout mouse model. This was attributed to deregulation of the COMPASS complex and activation of oncogenes MYC and RUNX3 (Zhu et al., 2014). The cells also had increased sensitivity to bromodomains and extra-terminal (BET) inhibitors that target a type of epigenetic “reader” protein. Collectively this data suggests opportunities to indirectly target KDMs by studying the rich network of histone eraser or reader proteins that KDMs interact with.

In contrast to loss of KDM6A its presence may also be associated with other cancer types such as cervical cancer, where it appeared necessary for HPVE7 expressing cells to survive and de-repress the cell cycle DNA replication inhibitor p21 (Soto et al., 2017). In addition, KDM6A has been shown to support the oncogenic function of the estrogen receptor in breast cancer (Kim et al., 2017; Taube et al., 2017; Xie et al., 2017). This suggests KDM6A may operate in a cell type dependant manner and further investigation is required to resolve the tumor suppressive vs oncogenic role in different types of cancer.

KDM6B also known as JMJD3 has been reported to induce expression of oncogenes of the RAS/RAF MAP kinase signaling pathway and is expressed on activated macrophages, purported to play a role in inflammation (Agger et al., 2009).

Initial studies showed that KDM6B expression was important in the progression and prognosis of colon cancer (Pereira et al., 2011; Tokunaga et al., 2016). KDM6B has been reported to be overexpressed in prostate cancer with its expression increasing incrementally as the diseases progresses (Daures et al., 2016). In addition, KDM6B was found to induce epithelial to mesenchymal transition (EMT) and metastasis in clear cell renal carcinoma *via* activation of EMT factor Slug (Li et al., 2015). KDM6B has been reported to be overexpressed in diffuse large B-cell lymphoma (DLBCL) and is associated with poor survival. When DLBCL cells were treated with a small molecule KDM6 inhibitor (GSK-J4) not only was KDM6B expression inhibited, the cells were sensitized to chemotherapy agents (Mathur et al., 2017). KDM6B is highly expressed in multiple myeloma cells and when KDM6B was knocked down, growth and survival of these cells was inhibited (Ohguchi et al., 2017). Recent studies found that KDM6B overexpression promoted ovarian cancer cell migration and invasion *via* modulation of transforming growth factor-β1 (TGF-β1) (Pinton et al., 2018; Liang et al., 2019). In contrast, KDM6B overexpression induced cell apoptosis in non-small cell lung cancer (NSCLC) *via* translocation of FOXO1 (Ma et al., 2015) indicating that the role of KDM6B may be cell type dependent.

KDM7A/JHDM1D can remove methyl groups from di and trimethylated lysines on histone H3K4 as well as methyl groups from H3K9 (Klose et al., 2006; Wen et al., 2010). KDM7A has been reported to play an important role in the regulation of neural differentiation in particular the regions of the brain (Huang et al., 2010; Tsukada et al., 2010) but is not well studied in cancer.

### KDM8 Demethylase

The most recently identified KDM is KDM8 also known as JMJD5 and demethylates H3K36me2 inducing gene activation. KDM8 was initially identified to play an important role in embryogenesis and stem cell renewal (Oh and Janknecht, 2012; Zhu et al., 2014) and can promote carcinogenesis. A study showed KDM8 overexpression induced the expression of cell cycle promoter gene cyclin D1, promoting cell proliferation in a breast cancer cells model *in vitro* and conversely, KDM8 knockdown inhibited cell growth (Hsia et al., 2010). In addition, KDM8 overexpression induces activation of AR transcriptional activity and promotes cell growth in prostate cancer *in vitro* and *in vivo* (Wang et al., 2019).

**FIGURE 3:**
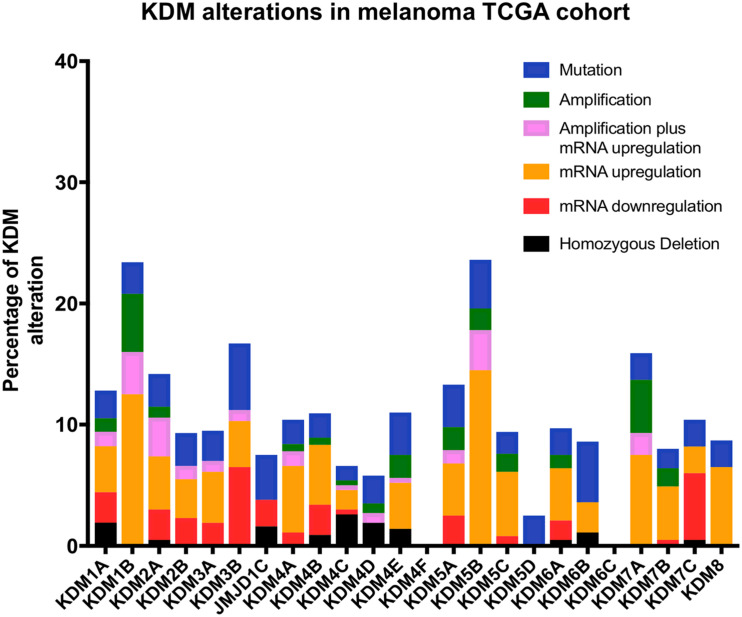
Bar graph showing the percentage and type of each KDM mutations obtained from the cancer genome atlas (TCGA) database. The data was obtained from a total of 472 patients with skin cutaneous melanoma (SKCM).

**FIGURE 4:**
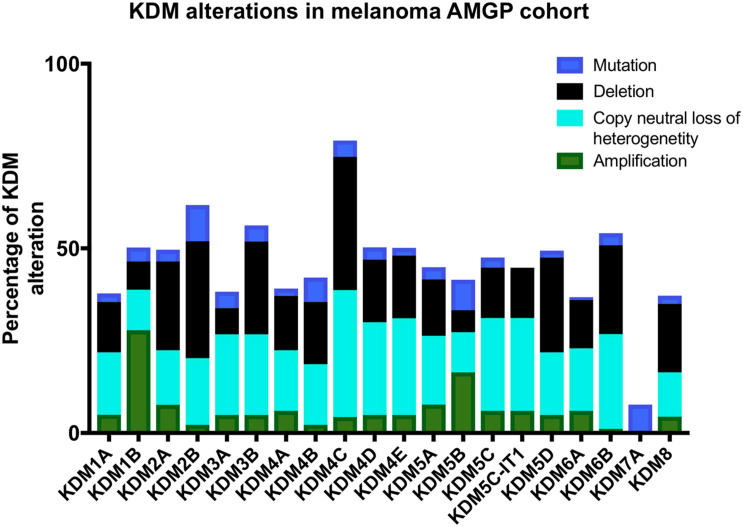
Bar graph showing the percentage of each and type of KDM alterations in 183 melanoma patients from the Australian melanoma genome project (AMGP).

Lysine demethylases have been implicated in a variety of cancers (summarized in Table 1) however, very few KDMs have been investigated in melanoma. We analyzed the percentage of mutation rates and types found in KDMs in the melanoma cohort obtained from the cancer genome atlas (TCGA) (Figure 3) and in a second independent dataset known as the Australian Melanoma Genome Project (AMGP) (Figure 4). We found that KDM1B and KDM5B exhibited the highest mutation rates in melanoma in the TCGA dataset. The majority of KDMs with the exception of KDM6C are upregulated in this dataset further emphasizing their importance in melanogenesis (Figure 3). KDM2B and KDM4C contained the highest number of alterations in the AMGP, however, this dataset does not include mRNA expression. The current knowledge and relevance of KDMs in melanoma will be discussed in the following section.

### Current Knowledge and Importance of KDMs in Melanogenesis

Melanoma is the most deadly type of skin cancer and is the most commonly diagnosed cancer in young Australians (AIHW, 2017). Analysis of two independent cohorts showed that KDM alterations are frequent in melanoma (Figures 3, 4) and thus warrant further investigation into their mechanistic role.

The most extensively studied KDM in melanoma is KDM5B. Studies found that KDM5B expression is higher in melanocytic nevi compared to advanced and metastatic melanomas (Roesch et al., 2005, 2010). High KDM5B expression was associated with a slow cycling population of melanoma cells that prolonged growth and self-renewal. An effect was observed on Notch signaling in that KDM5B suppressed the Notch ligand Jagged 1, causing less notch cleavage and a decrease in the expression of Notch target genes. The study suggests that KDM5B may have an important role in the maintenance of stem like progenitors that seed tumor progression and metastasis in melanoma.

KDM6B was found to be essential for melanoma tumor growth and metastasis (Park et al., 2016). KDM6B could activate NF-kB and bone morphogenic protein signaling promoting melanoma growth and progression.

Another recent study showed that H3K9 demethylases (which include KDM3B) can disable senescence, allowing *ras/braf* mutant melanoma development and progression. This was reversed when treated with H3K9 inhibitors *in vitro* and *in vivo* (Yu et al., 2018). The main role of KDM6A/B may be as an antagonist of EZH2 which has been implicated in the growth and progression in melanoma. Early studies found that elevated EZH2 expression was associated with poor survival (Bachmann et al., 2006; McHugh et al., 2007). A melanoma EZH2 mouse model showed that melanocyte specific loss of EZH2, or treatment with an EZH2 inhibitor, abolished the spread of metastatic melanoma (Zingg et al., 2015). EZH2 was able to induce resistance to immunotherapy treatments anti-CTLA-4 and IL-2 in a melanoma mouse model and when inactivated, reversed this resistance (Zingg et al., 2017). A recent study also found that EZH2 induced loss of primary cilia, enhanced Wnt signaling and promoted melanoma metastasis (Zingg et al., 2018). Collectively these studies show that EZH2 has an important role in the progression of melanoma, therefore its only known antagonist, KDM6A/B, is likely to be of equal importance and warrants exploration. It is also unclear what role the COMPASS complex which contains KDM6A may have in melanoma progression. Importantly it is not clear what role these complexes may have in determining the sensitivity or resistance to inhibitors of EZH2 and KDM6A/B.

### Potential KDM Sex Specific Roles in Melanoma?

There is a striking and unexplained predominance for males to be diagnosed and die from cancer, including melanoma, compared to females (Joosse et al., 2013; Clocchiatti et al., 2016; Enninga et al., 2017). It is postulated that this is due to particular X-linked genes that escape X-inactivation also known as “escape from X-inactivation tumor suppressor” (EXITS) genes (Dunford et al., 2017). This means that females express two copies of EXITS genes compared to males, effectively doubling the amount of tumor suppressive function. One of these KDMS identified in this category was KDM6A. Our recent study showed that KDM6A had a significant prognostic effect in female melanoma patients inducing better overall survival (Emran et al., 2020). Our TCGA gene set enrichment analysis (GSEA) suggests that high KDM6A level associated with upregulation of several immune related pathways like IFN-gamma which may help anti-tumor immunity and survival advantage in female melanoma patients compared to male (Emran et al., 2020).

Other studies support KDM6A in having sex-specific roles in normal biology and cancer. An early study showed that KDM6A expression was significantly higher in the brains and organs of female mice compared to male mice (Xu et al., 2008). In the context of cancer, a study showed that KDM6A had a gender-specific, tumor suppressive effect in T-cell acute lymphoblastic leukemia (T-ALL). The study showed that KDM6A is frequently mutated in male T-ALL patients and KDM6A expression is significantly lower compared to females T-ALL patients (Van der Meulen et al., 2015). In addition, loss of KDM6A expression in female mice induced poorer survival by downregulation of T-ALL associated tumor suppressor genes and upregulation of T-ALL associated oncogenes (Van der Meulen et al., 2015). Loss of KDM6A expression in female mice exhibited a squamous-like, malignant phenotype *via* activation of oncogenes MYC and RUNX3 in a pancreatic cancer mouse model (Andricovich et al., 2018).

UTY also known as KDM6C is the male equivalent of KDM6A/UTX and is expressed on the Y chromosome but importantly, has significantly reduced H3K27me3 demethylation activity compared to KDM6A, due to the substitution of important amino acids in the Jumonji C domain (Walport et al., 2014).

A study in pancreatic cancer found that concurrent loss of UTY and KDM6A in male patients was associated with a more malignant phenotype and poorer and prognosis (Andricovich et al., 2018). In addition, male mice that expressed UTY and female mice with heterozygous KDM6A expression exhibited a less aggressive pancreatic cancer phenotype, while male and female mice with no UTY or KDM6A exhibited a more aggressive malignant phenotype (Andricovich et al., 2018). Hence this study suggests that KDM6A and UTY have tumor-suppressive roles in pancreatic cancer and that sex-specific mechanism should be investigated in melanoma and other cancers.

## KDM Inhibitors

Numerous KDM inhibitors are showing promise in targeting KDMs in certain types of cancers in both preclinical and clinical studies. A summary of inhibitors is provided below and in Table 2, grouped by KDM family.

**TABLE 2:** Current KDM inhibitors in clinical and preclinical trials.

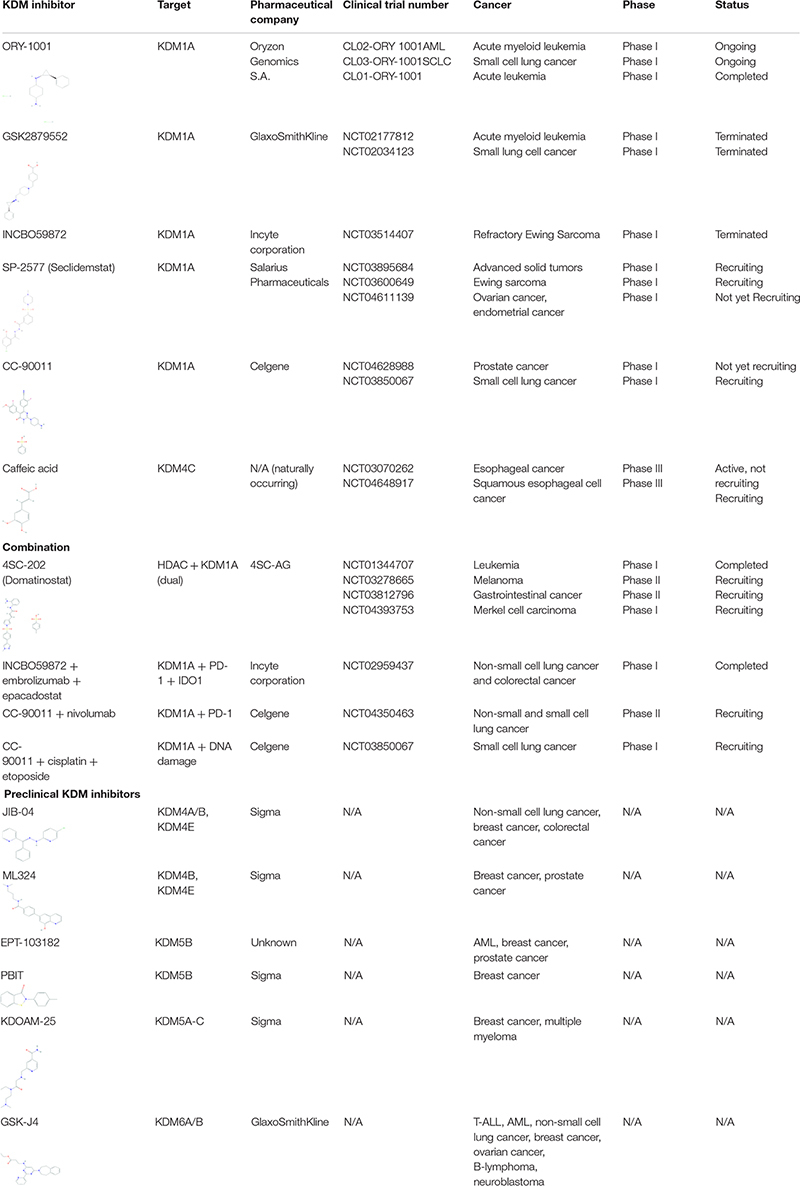

### KDM1

There have been numerous irreversible and reversible inhibitors that have been developed and tested preclinically and in clinical trials that target the KDM1 or LSD family. These include tranylcypromine derived KDM1A inhibitors that have comprehensively described elsewhere (Fang et al., 2019) and summarized below.

#### ORY-1001

ORY-1001 has been reported to induce expression of differentiation markers in mixed lineage leukemia (MLL) cells as well as reducing tumor growth in an acute myeloid leukemia (AML) mouse model and possesses good oral bioavailability (Harris et al., 2012; Maes et al., 2013, 2015). ORY-1001 has also been shown to suppress growth in lung cancer *in vitro* (Lu et al., 2018). ORY-1001 is currently being tested in phase I and II clinical trials in patients with relapsed and/or refractory AML and small cell lung cancer (SCLC) (Register ECT, 2018).

#### GSK2879552

Another selective KDM1A inhibitor is GSK2879552, tested in phase I clinical trials in SCLC. A study found that GSK2879552 promotes differentiation in AML cells and when SCLC cells were treated, proliferation was reduced *in vitro*. In mouse models of AML and SCLC, GSK2879552 prolonged survival (Dhanak and Jackson, 2014; Mohammed et al., 2014). However, clinical trials in patients with AML and SCLC and were terminated due to adverse side effects (Table 2).

#### INCB059872

Initially, the KDM1A inhibitor known as INCB059872 treatment was shown to induce cell differentiation in progenitor cells in AML (Johnston et al., 2020), prostate cancer and Ewing sarcoma (Fang et al., 2019). However, INCB059872 was tested in a clinical trial in patients with Ewing sarcoma and terminated due to recruitment issues, this inhibitor in conjunction with an anti-PD-1 checkpoint inhibitor is currently being tested in patients with colorectal and lung cancer (Table 2).

#### 4SC-202

Another strategy involves dual inhibition of both histone deacetylases (HDAC) and KDM1A with 4SC-202 which targets HDAC1, 2, 3, and KDM1A and has been shown to inhibit the stem-related properties of cancer cells reducing their viability (Henning et al., 2010). A phase I clinical study has been conducted for 4SC-202 in patients with advanced leukemia, found to possess anti-cancer activity and to be well-tolerated in patients (von Tresckow et al., 2014, 2019). Another study found that treatment with 4SC-202 significantly increased the survival of mice that had AML tumors without any toxicity effects (Fiskus et al., 2014). Currently, there are clinical trials being undertaken that are testing the effect of 4SC-202 in patients with melanoma, merkel cell carcinoma and gastrointestinal cancer (Table 2).

#### SP-2577

SP-2577 reversibly inhibits KDM1A demethylation and has been recently found to promote anti tumor immunity in mutated ovarian cancer cells *in vitro* and has also been found to inhibit growth in Ewing Sarcoma xenografts (Salarius Pharmaceuticals, Inc., 2020; Soldi et al., 2020). Currently SP-2577 is being tested in patients with Ewing sarcoma, ovarian and endometrial cancers (Table 2).

#### CC-90011

The reversible KDM1A inhibitor CC-90011 has been found to induce cellular differentiation exhibits anti-tumor efficacy *in vitro* and *in vivo* in AML and SCLC (Kanouni et al., 2020). CC-90011 was also tested in patients with hematological cancers, showing robust anti-cancer properties and good tolerability in patients (Hollebecque et al., 2021). Currently, CC-90011 is being tested in clinical trials in patients with prostate and SCLC (Table 2).

### KDM4

#### Caffeic Acid

The most prominent KDM4 inhibitor is caffeic acid, a naturally occurring compound found in various sources including eucalyptus globus (Santos et al., 2011). Caffeic acid has been reported to mainly target KDM4C (Nielsen et al., 2012) and displays potent anti-cancer activity against esophageal cancer *in vitro* and *in vivo* (Hirose et al., 1998). Currently, caffeic acid is being tested in a clinical trial in patients with esophageal cancer (Table 2).

#### JIB-04

The most advanced preclinical KDM4 inhibitor is JIB-04 which targets KDM4A, KDM4B, and KDM4E and can inhibit growth and reduce tumor burden in non-small cell lung cancer (NSCLC) and breast cancer *in vitro* and *in vivo* (Wang et al., 2013). In addition, JIB-04 treatment reduced colony formation, growth and migration *in vitro* and reduced tumorigenic activity in a colorectal cancer model *in vivo*. The mechanism has been attributed to downregulating genes of the Wnt signaling pathway which are essential for promoting carcinogenesis (Kim et al., 2018). JIB-04 is yet to be tested in clinical trials.

#### ML324

Another KDM4 inhibitor in the toolbox is ML324 which targets KDM4B and KDM4E (Rai et al., 2010). A study reported that ML324 treatment reduced tumor volume and growth in a triple negative breast cancer mouse model (Wang W. et al., 2018) and also inhibits proliferation in prostate cancer *in vitro* and *in vivo* (Carter et al., 2017). ML324 is yet to be tested in clinical trials.

### KDM5

#### EPT-103182

The most advanced KDM5 inhibitor is EPT-103182. This small molecule compound targets KDM5B which has been shown to have an anti-proliferative effect in hematological and solid cancer cell lines as well as inhibiting tumor growth in a dose-dependent manner in xenograft models (Hancock et al., 2015; Maes et al., 2015). This inhibitor has yet to be tested in clinical trials.

#### PBIT

Another recently identified KDM5 inhibitor is PBIT, shown to specially target and inhibit KDM5B (Sayegh et al., 2013). In the context of cancer, PBIT treatment inhibited proliferation of breast cancer by derepression and upregulation of the tumor suppressor HEXIMI *in vitro* (Montano et al., 2019). PBIT is yet to be tested in clinical trials.

#### KDOAM-25

KDOAM-25 has been shown to target and inhibit KDM5A-C demethylases (a pan-KDM5 family inhibitor), but especially KDM5B (Tumber et al., 2017). KDOAM-25 treatment has been shown to reduce proliferation and growth in breast cancer and multiple myeloma which highly expressed KDM5B (Tumber et al., 2017; Montano et al., 2019). KDOAM-25 is yet to be tested in clinical trials.

### KDM6

#### GSK-J4

Other inhibitors which target JMJC domain containing KDMs are known as GSK-J4 that targets KDM6A and KDM6B, shown to reduce the production of pro-inflammatory cytokines by human macrophages, although there have been questions in regards to its specificity (Kruidenier et al., 2012; Heinemann et al., 2014). Over the last few years, numerous preclinical studies have demonstrated that GSK-J4 could be a potential therapeutic for certain cancers. GSK-J4 treatment was initially shown to be effective in inhibiting cell growth and inducing cell cycle arrest and apoptosis in primary human T-cell acute lymphoblastic leukemia (T-ALL) lines (Ntziachristos et al., 2014). A recent study found that GSK-J4 treatment inhibited cell proliferation and colony forming ability of acute myeloid leukemia (AML) cell lines and inhibited tumor growth in an AML xenograft mouse model (Li et al., 2018).

Other studies have shown that GSK-J4 treatment inhibited proliferation of castration-resistant prostate cancer cells by inhibiting AR–driven transcription and can also inhibit proliferation in glioma cells in a dose-dependent manner *in vitro* (Morozov et al., 2017; Sui et al., 2017). A study also showed that GSK-J4 suppressed the ability of breast cancer and ovarian cancer stem cells to proliferate and grow (Sakaki et al., 2015; Yan et al., 2017). GSK-J4 in combination with an anti-diabetic drug metformin induced cell death and inhibited cell growth in non-small cell lung cancer (NSCLC) cell lines (Watarai et al., 2016) and another study found GSK-J4 treatment sensitized diffuse large B-lymphoma cells to chemotherapy drugs (Mathur et al., 2017). GSK-J4 inhibited cell growth and upregulated apoptosis markers in neuroblastoma cell lines and inhibited tumor growth in an *in vivo* neuroblastoma model (Lochmann et al., 2018). GSK-J4 is yet to be tested in clinical trials.

## Concluding Remarks

Dysregulation of lysine methyl demethylases due to genetic changes or aberrant signaling are associated with a number of different cancers. The amine oxidase group of KDMs appear to mediate their effects by action on the H3K4 and H3K9 histone marks but in addition by interactions with the P53 tumor suppressor and E2F transcription factors regulating cell division.

The 2-oxoglutarate dependent oxygenases are the largest group of KDMs and mediate demethylation of histones at several important activating or repressive marks. Their effects in cancers range from tumor suppression to promotion and growth of cancers. Additional roles in immune responses against cancer have been revealed for KDM5C and KDM6A in survival studies on human melanoma patients and may be linked to higher expression from X linked chromosomes. Several KDMs are part of protein complexes like the COMPASS complex that contains KDM6A as well as methyl transferases MLL2 and MLL3 that regulate gene transcription in certain cancers.

The role of KDMs in cancer have identified them as potential therapeutic targets and a wide range of pharmacological agents have been developed. Given the complex interactions of KDMs with other epigenetic regulators it is not surprising that drugs targeting KDMs are yet to enter clinical practice and this remains a focus of future research.

## Author Contributions

GP-M, AE, and JT: design and structure of review. GP-M and AE: figures. GP-M: tables. GP-M, AE, PH, and JT: editing the manuscript. All authors contributed to the article and approved the submitted version.

## Conflict of Interest

The authors declare that the research was conducted in the absence of any commercial or financial relationships that could be construed as a potential conflict of interest.
